# Deep fake detection and classification using error-level analysis and deep learning

**DOI:** 10.1038/s41598-023-34629-3

**Published:** 2023-05-08

**Authors:** Rimsha Rafique, Rahma Gantassi, Rashid Amin, Jaroslav Frnda, Aida Mustapha, Asma Hassan Alshehri

**Affiliations:** 1 Department of Computer Science, University of Engineering and Technology, Taxila, Pakistan 47050; 2 Department of Electrical Engineering, Chonnam National University, Gwangju, 61186 South Korea; 3 Department of Computer Science, University of Chakwal, Chakwal, 48800 Pakistan; 4 Department of Quantitative Methods and Economic Informatics, Faculty of Operation and Economics of Transport and Communications, University of Zilina, 01026 Zilina, Slovakia; 5 Department of Telecommunications, Faculty of Electrical Engineering and Computer Science, VSB Technical University of Ostrava, 70800 Ostrava, Czech Republic; 6 Faculty of Applied Sciences and Technology, Universiti Tun Hussein Onn Malaysia, KM1 Jalan Pagoh, 84600 Pagoh, Johor, Malaysia; 7 Durma College of Science and Humanities, Shaqra University, Shaqra, 11961 Saudi Arabia

**Keywords:** Energy science and technology, Mathematics and computing

## Abstract

Due to the wide availability of easy-to-access content on social media, along with the advanced tools and inexpensive computing infrastructure, has made it very easy for people to produce deep fakes that can cause to spread disinformation and hoaxes. This rapid advancement can cause panic and chaos as anyone can easily create propaganda using these technologies. Hence, a robust system to differentiate between real and fake content has become crucial in this age of social media. This paper proposes an automated method to classify deep fake images by employing Deep Learning and Machine Learning based methodologies. Traditional Machine Learning (ML) based systems employing handcrafted feature extraction fail to capture more complex patterns that are poorly understood or easily represented using simple features. These systems cannot generalize well to unseen data. Moreover, these systems are sensitive to noise or variations in the data, which can reduce their performance. Hence, these problems can limit their usefulness in real-world applications where the data constantly evolves. The proposed framework initially performs an Error Level Analysis of the image to determine if the image has been modified. This image is then supplied to Convolutional Neural Networks for deep feature extraction. The resultant feature vectors are then classified via Support Vector Machines and K-Nearest Neighbors by performing hyper-parameter optimization. The proposed method achieved the highest accuracy of 89.5% via Residual Network and K-Nearest Neighbor. The results prove the efficiency and robustness of the proposed technique; hence, it can be used to detect deep fake images and reduce the potential threat of slander and propaganda.

## Introduction

In the last decade, social media content such as photographs and movies has grown exponentially online due to inexpensive devices such as smartphones, cameras, and computers. The rise in social media applications has enabled people to quickly share this content across the platforms, drastically increasing online content, and providing easy access. At the same time, we have seen enormous progress in complex yet efficient machine learning (ML) and Deep Learning (DL) algorithms that can be deployed for manipulating audiovisual content to disseminate misinformation and damage the reputation of people online. We now live in such times where spreading disinformation can be easily used to sway peoples’ opinions and can be used in election manipulation or defamation of any individual. Deep fake creation has evolved dramatically in recent years, and it might be used to spread disinformation worldwide, posing a serious threat soon. Deep fakes are synthesized audio and video content generated via AI algorithms. Using videos as evidence in legal disputes and criminal court cases is standard practice. The authenticity and integrity of any video submitted as evidence must be established. Especially when deep fake generation becomes more complex, this is anticipated to become a difficult task.

The following categories of deep fake videos exist: face-swap, synthesis, and manipulation of facial features. In face-swap deep fakes, a person's face is swapped with that of the source person to create a fake video to target a person for the activities they have not committed^1^, which can tarnish the reputation of the person^2^. In another type of deep fake called lip-synching, the target person’s lips are manipulated to alter the movements according to a certain audio track. The purpose of lip-syncing is to simulate the victim's attacker's voice by having someone talk in that voice. With puppet-master, deep fakes are produced by imitating the target's facial expressions, eye movements, and head movements. Using fictitious profiles, this is done to propagate false information on social media. Last but not least, deep audio fakes or voice cloning is used to manipulate an individual's voice that associates something with the speaker they haven’t said in actual^1,3^.

The importance of discovering the truth in the digital realm has therefore increased. Dealing with deep fakes is significantly more difficult because they are mostly utilized for harmful objectives and virtually anyone can now produce deep fakes utilizing the tools already available. Many different strategies have been put out so far to find deep fakes. Since most are also based on deep learning, a conflict between bad and good deep learning applications has developed^4^. Hence, to solve this problem, the United States Defense Advanced Research Projects Agency (DARPA) launched a media forensics research plan to develop fake digital media detection methods^5^. Moreover, in collaboration with Microsoft, Facebook also announced an AI-based deep fake detection challenge to prevent deep fakes from being used to deceive viewers^6^.

Over the past few years, several researchers have explored Machine Learning and Deep Learning (DL) areas to detect deep fakes from audiovisual media. The ML-based algorithms use labor-intensive and erroneous manual feature extraction before the classification phase. As a result, the performance of these systems is unstable when dealing with bigger databases. However, DL algorithms automatically carry out these tasks, which have proven tremendously helpful in various applications, including deep fake detection. Convolutional neural network (CNN), one of the most prominent DL models, is frequently used due to its state-of-the-art performance that automatically extracts low-level and high-level features from the database. Hence, these methods have drawn the researcher’s interest in scientists across the globe^7^.

Despite substantial research on the subject of deep fakes detection, there is always potential for improvement in terms of efficiency and efficacy. It may be noted that the deep fake generation techniques are improving quickly, thus resulting in increasingly challenging datasets on which previous techniques may not perform effectively. The motivation behind developing automated DL based deep fake detection systems is to mitigate the potential harm caused by deep fake technology. Deep fake content can deceive and manipulate people, leading to serious consequences, such as political unrest, financial fraud, and reputational damage. The development such systems can have significant positive impacts on various industries and fields. These systems also improve the trust and reliability of media and online content. As deep fake technology becomes more sophisticated and accessible, it is important to have reliable tools to distinguish between real and fake content. Hence, developing a robust system to detect deep fakes from media has become very necessary in this age of social media. This paper is a continuation of to study provided by Rimsha et al.^8^. The paper compares the performance of CNN architectures such as AlexNet and VGG16 to detect if the image is real of has been digitally altered. The main contributions of this study are as follows:

- In this study, we propose a novel deep fake detection and classification method employing DL and ML-based methods.
- The proposed framework preprocesses the image by resizing it according to CNN’s input layer and then performing Error Level Analysis to find any digital manipulation on a pixel level.
- The resultant ELA image is supplied to Convolutional Neural Networks, i.e., GoogLeNet, ResNet18 and SqueezeNet, for deep feature extraction.
- Extensive experiments are conducted to find the optimal hyper-parameter setting by hyper-parameter tuning.
- The performance of the proposed technique is evaluated on the publically available dataset for deep fake detection

## Related work

The first ever deep fake was developed in 1860, when a portrait of southern leader John Calhoun was expertly altered for propaganda by swapping his head out for the US President. These manipulations are typically done by splicing, painting, and copy-moving the items inside or between two photos. The appropriate post-processing processes are then used to enhance the visual appeal, scale, and perspective coherence. These steps include scaling, rotating, and color modification^9,10^. A range of automated procedures for digital manipulation with improved semantic consistency are now available in addition to these conventional methods of manipulation due to developments in computer graphics and ML/DL techniques. Modifications in digital media have become relatively affordable due to widely available software for developing such content. The manipulation is in digital media is increasing at a very fast pace which requires development of such algorithms to robustly detect and analyze such content to find the difference between right and wrong^11–13^.

Despite being a relatively new technology, deep fake has been the topic of investigation. In recent years, there had been a considerable increase in deep fake articles towards the end of 2020. Due to the advent of ML and DL-based techniques, many researchers have developed automated algorithms to detect deep fakes from audiovisual content. These techniques have helped in finding out the real and fake content easily. Deep learning is well renowned for its ability to represent complicated and high-dimensional data^11,14^. Matern et al.^15^ employed detected deep fakes from Face Forensics dataset using Multilayered perceptron (MLP) with an AUC of 0.85. However, the study considers facial images with open eyes only. Agarwal et al.^16^ extracted features using Open Face 2 toolkit and performed classification via SVM. The system obtained 93% AUC; however, the system provides incorrect results when a person is not facing camera. The authors in Ciftci et al.^17^ extracted medical signal features and performed classification via CNN with 97% accuracy. However, the system is computationally complex due to a very large feature vector. In their study, Yang et al.^18^ extracted 68-D facial landmarks using DLib and classified these features via SVM. The system obtained 89% ROC. However, the system is not robust to blurred and requires a preprocessing stage. Rossle et al.^19^ employed SVM + CNN for feature classification and a Co-Occurrence matrix for feature extraction. The system attained 90.29% accuracy on Face Forensics dataset. However, the system provides poor results on compressed videos. McCloskey et al.^20^ developed a deep fake detector by using the dissimilarity of colors between real camera and synthesized and real image samples. The SVM classifier was trained on color based features from the input samples. However, the system may struggle on non-preprocessed and blurry images.

A Hybrid Multitask Learning Framework with a Fire Hawk Optimizer for Arabic Fake News Detection aims to address the issue of identifying fake news in the Arabic language. The study proposes a hybrid approach that leverages the power of multiple tasks to detect fake news more accurately and efficiently. The framework uses a combination of three tasks, namely sentence classification, stance detection, and relevance prediction, to determine the authenticity of the news article. The study also suggests the use of the Fire Hawk Optimizer algorithm, a nature-inspired optimization algorithm, to fine-tune the parameters of the framework. This helps to improve the accuracy of the model and achieve better performance. The Fire Hawk Optimizer is an efficient and robust algorithm that is inspired by the hunting behavior of hawks. It uses a global and local search strategy to search for the optimal solution^21^. The authors in^22^ propose a Convolution Vision Transformer (CVT) architecture that differs from CNN in that it relies on a combination of attention mechanisms and convolution operations, making it more effective in recognizing patterns within images.The CVT architecture consists of multi-head self-attention and multi-layer perceptron (MLP) layers. The self-attention layer learns to focus on critical regions of the input image without the need for convolution operations, while the MLP layer helps to extract features from these regions. The extracted features are then forwarded to the output layer to make the final classification decision. However, the system is computationally expensive due to deep architecture. Guarnera et al.^23^ identified deep fake images using Expectation Maximization for extracting features and SVM, KNN, LDA as classification methods. However, the system fails in recognizing compressed images. Nguyen et al.^24^ proposed a CNN based architecture to detect deep fake content and obtained 83.7% accuracy on Face Forensics dataset. However, the system is unable to generalize well on unseen cases. Khalil et al.^25^ employed Local Binary Patterns (LBP) for feature extraction and CNN and Capsule Network for deep fake detection. The models were trained on Deep Fake Detection Challenge-Preview dataset and tested on DFDC-Preview and Celeb- DF datasets. A deep fake approach developed by Afchar et al.^26^ employed MesoInception-4 and achieved 81.3% True Positive Rate via Face Forensics dataset.

However, the system requires preprocessing before feature extraction and classification. Hence, results in a low overall performance on low-quality videos. Wang et al.^27^ evaluated the performance of Residual Networks on deep fake classification. The authors employed ResNet and ResNeXt, on videos from Face forensics dataset. In another study by Stehouwer et al.^28^, the authors presented a CNN based approach for deep fake content detection that achieved 99% overall accuracy on Diverse Fake Face Dataset. However, the system is computationally expensive due to a very large size feature vector. Despite significant progress, existing DL algorithms are computationally expensive to train and require high-end GPUs or specialized hardware. This can make it difficult for researchers and organizations with limited resources to develop and deploy deep learning models. Moreover, some of the existing DL algorithms are prone to overfitting, which occurs when the model becomes too complex and learns to memorize the training data rather than learning generalizable patterns. This can result in poor performance on new, unseen data. The limitations in the current methodologies prove there is still a need to develop a robust and efficient deep fake detection and classification method using ML and DL based approaches.

## Proposed methodology

**Figure 1:**
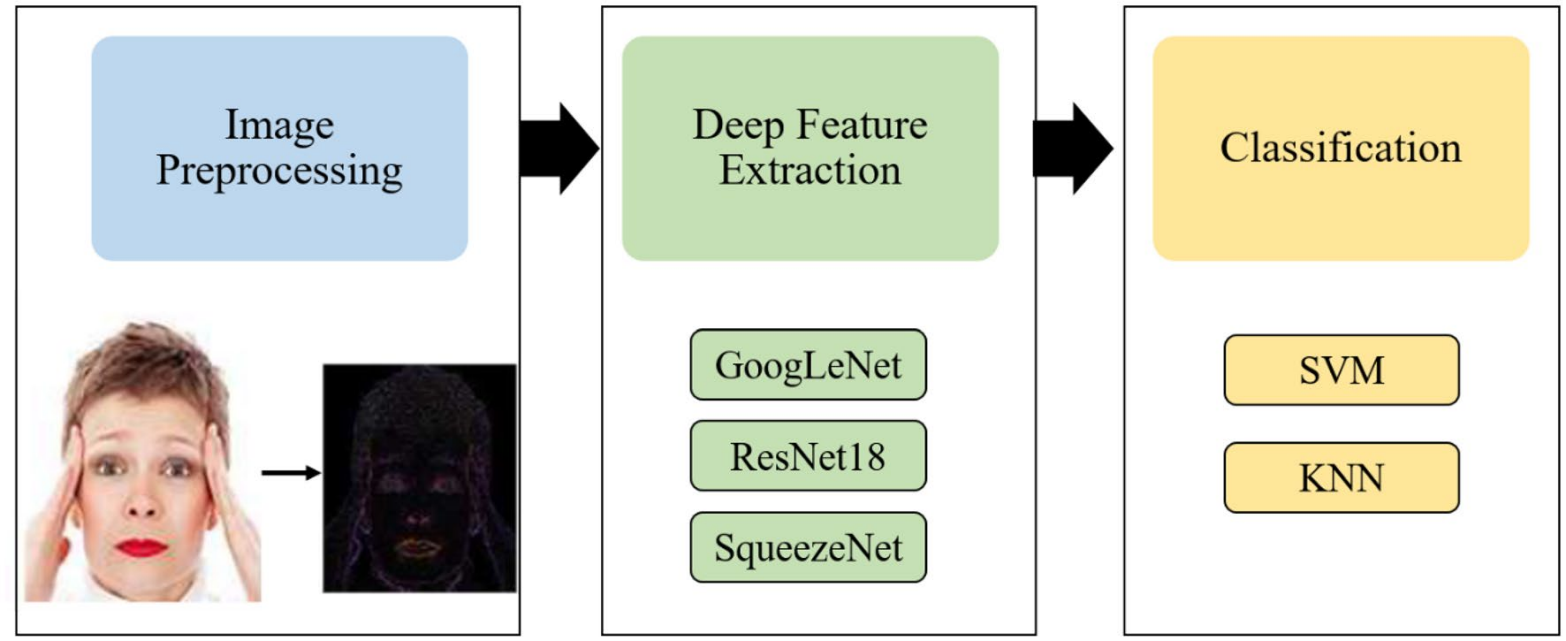
Workflow diagram of the proposed method.

This section discusses the proposed workflow employed for deep fakes detection. The workflow diagram of our proposed framework is illustrated in Fig. 1. The proposed system comprises of three core steps (i) image preprocessing by resizing the image according to CNN’s input layer and then generating Error Level Analysis of the image to determine pixel level alterations (ii) deep feature extraction via CNN architectures (iii) classification via SVM and KNN by performing hyper-parameter optimization.

### (i) Error level analysis

Error level analysis, also known as ELA, is a forensic technique used to identify image segments with varying compression levels. By measuring these compression levels, the method determines if an image has undergone digital editing. This technique works best on .JPG images as in that case, the entire image pixels should have roughly the same compression levels and may vary in case of tampering^29,30^.

JPEG (Joint Photographic Experts Group) is a technique for the lossy compression of digital images. A data compression algorithm discards (loses) some of the data to compress it. The compression level could be used as an acceptable compromise between image size and image quality. Typically, the JPEG compression ratio is 10:1. The JPEG technique uses 8 × 8 pixel image grids independently compressed. Any matrices larger than 8 × 8 are more difficult to manipulate theoretically or are not supported by the hardware, whereas any matrices smaller than 8 × 8 lack sufficient information.

Consequently, the compressed images are of poor quality. All 8 × 8 grids for unaltered images should have a same error level, allowing for the resave of the image. Given that uniformly distributed faults are throughout the image, each square should deteriorate roughly at the same pace. The altered grid in a modified image should have a higher error potential than the rest^31^.

**Figure 2:**
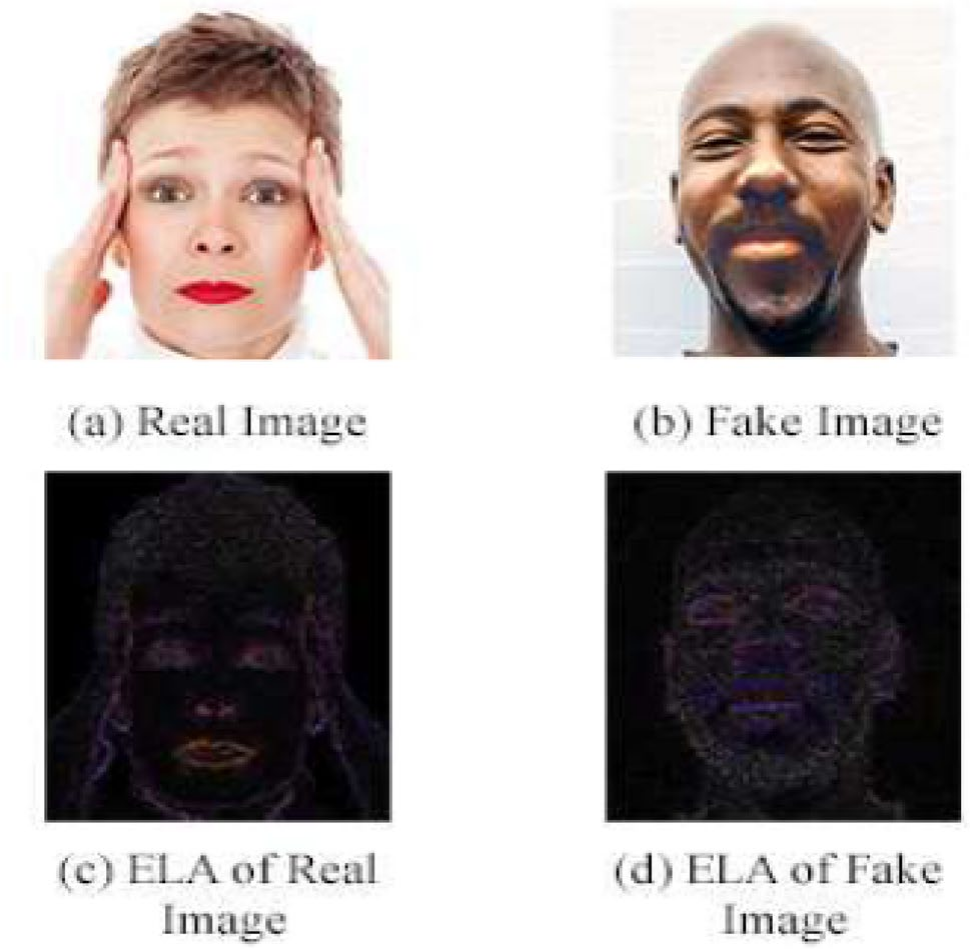
Result of ELA on dataset images.

ELA. The image is resaved with 95% error rate, and the difference between the two images is computed. This technique determines if there is any change in cells by checking whether the pixels are at their local minima^8,32^. This helps determine whether there is any digital tampering in the database. The ELA is computed on our database, as shown in Fig. 2.

### (ii) Feature extraction using convolutional neural networks

**Figure 3:**
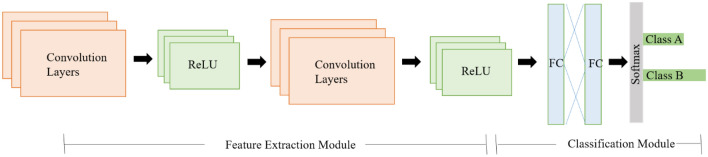
General CNN architecture.

The discovery of CNN has raised its popularity among academics and motivated them to work through difficult problems that they had previously given up on. Researchers have designed several CNN designs in recent years to deal with multiple challenges in various research fields, including deep fake detection. The general architecture of CNN as shown in Fig. 3, is usually made up of many layers stacked on top of one another. The architecture of CNN consists of a feature extraction module composed of convolutional layers to learn the features and pooling layers reduce image dimensionality. Secondly, it consists of a module comprising a fully connected (FC) layer to classify an image^33,34^.

The image is input using the input layer passed down to convolution for deep feature extraction. This layer learns the visual features from the image by preserving the relationship between its pixels. This mathematical calculation is performed on an image matrix using filter/kernel of the specified size^35^. The max-pooling layer reduces the image dimensions. This process helps increase the training speed and reduce the computational load for the next stages^36^. Some networks might include normalization layers, i.e., batch normalization or dropout layer. Batch normalization layer stabilizes the network training performance by performing standardization operations on the input to mini-batches. Whereas, the dropout layer randomly drops some nodes to reduce the network complexity, increasing the network performance^37,38^. The last layers of the CNN include an FC layer with a softmax probability function. FC layer stores all the features extracted from the previous phases. These features are then supplied to classifiers for image classification^38^. Since CNN architectures can extract significant features without any human involvement, hence, we used pre-trained CNNs such as GoogLeNet^39^, ResNet18^31^, and SqueezeNet^40^ in this study. It may be noted that developing and training a deep learning architecture from scratch is not only a time-consuming task but requires resources for computation; hence we use pre-trained CNN architectures as deep feature extractors in our proposed framework.

Microsoft introduced Residual Network (ResNet) architecture in 2015 that consists of several Convolution Layers of kernel size 3 × 3, an FC layer followed by an additional softmax layer for classification. Because they use shortcut connections that skip one or more levels, residual networks are efficient and low in computational cost^41^. Instead of anticipating that every layer stack will instantly match a specified underlying mapping, the layers fit a residual mapping. As a result of the resulting outputs being added to those of the stacked layers, these fast connections reduce loss of value during training. This functionality also aids in training the algorithm considerably faster than conventional CNNs.

**Figure 4:**
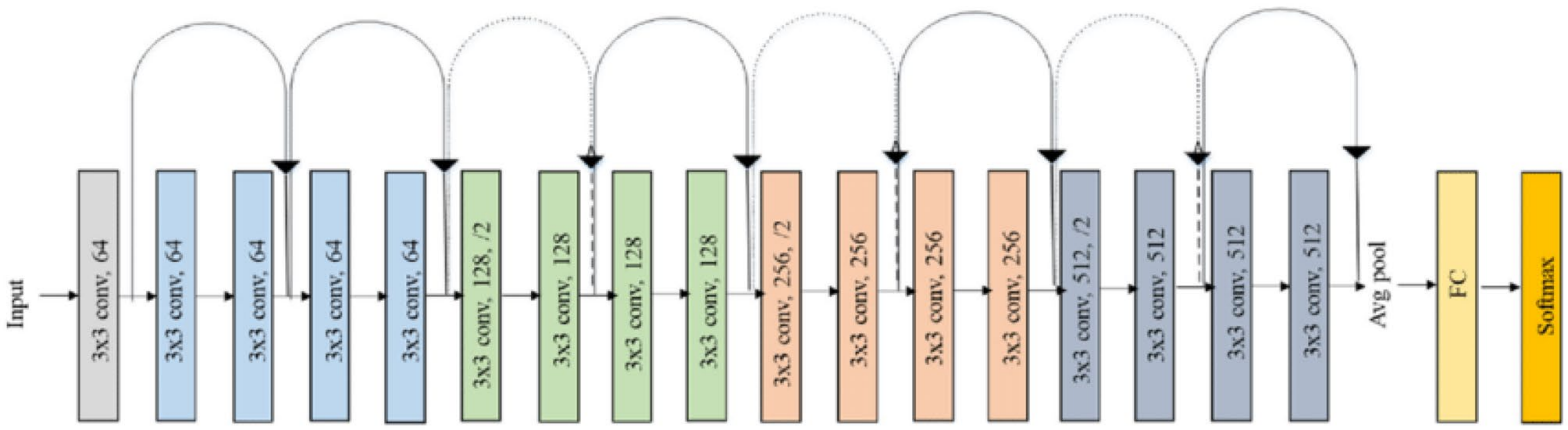
ResNet18 architecture^44^.

Furthermore, this mapping has no parameters because it transfers the output to the next layer. The ResNet architecture outperformed other CNNs by achieving the lowest top 5% error rate in a classification job, which is 3.57%^31,42^. The architecture of ResNet50 is shown in Fig. 4^43^.

SqueezNet was developed by researchers at UC Berkeley and Stanford University that is a very lightweight and small architecture. The smaller CNN architectures are useful as they require less communication across servers in distributed training. Moreover, these CNNs also train faster and require less memory, hence are not computationally expensive compared to conventional deep CNNs. By modifying the architecture, the researchers claim that SqueezeNet can achieve AlexNet level accuracy via a smaller CNN^45^. Because an 1 × 1 filter contains 9× fewer parameters than a 3 × 3 filter, the 3 × 3 filters in these modifications have been replaced with 1 × 1 filters. Furthermore, the number of input channels is reduced to 3 × 3 filters via squeeze layers, which lowers the overall number of parameters.

**Figure 5:**
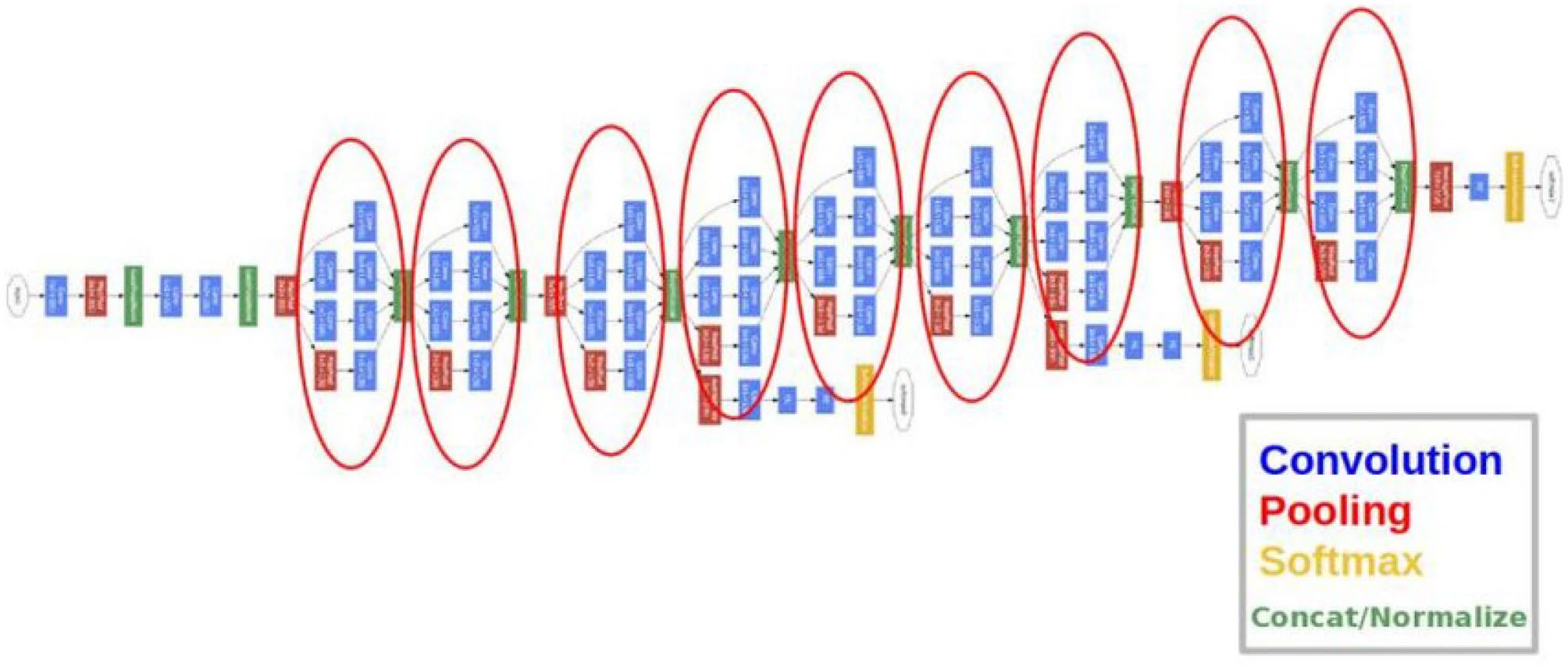
GoogLeNet architecture^46^.

Last but not least, the downsampling is carried out very late in the network so the convolution layers’ large activation maps which is said to increase classification accuracy^40^. Developed by Google researchers, GoogLeNet is a 22-layer deep convolutional neural network that uses a 1 × 1 convolution filter size, global average pooling and an input size of 224 × 224 × 3. The architecture of GoogLeNet is shown in Fig. 5. To increase the depth of the network architecture, the convolution filter size is reduced to 1 × 1. Additionally, the network uses global average pooling towards the end of the architecture, which inputs a 7 × 7 feature map and averages it to an 1 × 1 feature map. This helps reduce trainable parameters and enhances the system's performance. A dropout regularization of 0.7 is also used in the architecture, and the features are stored in an FC layer^39^.

CNNs extract features from images hierarchically using convolutional, pooling, and fully connected layers. The features extracted by CNNs can be broadly classified into two categories: low-level features and high-level features. Low-level features include edges, corners, and intensity variations. CNNs can detect edges by convolving the input image with a filter that highlights the edges in the image. They can also detect corners by convolving the input image with a filter that highlights the corners. Morever, CNNs can extract color features by convolving the input image with filters that highlight specific colors. On the other hand, high-level features include texture, objects, and contextual and hierarchical features. Textures from images are detected by convolving the input image with filters that highlight different textures. The CNNs detect objects by convolving the input image with filters highlighting different shapes. Whereas, contextual features are extracted by considering the relationships between different objects in the image. Finally, the CNNs can learn to extract hierarchical features by stacking multiple convolutional layers on top of each other. The lower layers extract low-level features, while the higher layers extract high-level features.

### (iii) Classification via support vector machines and k-nearest neighbors

**Figure 6:**
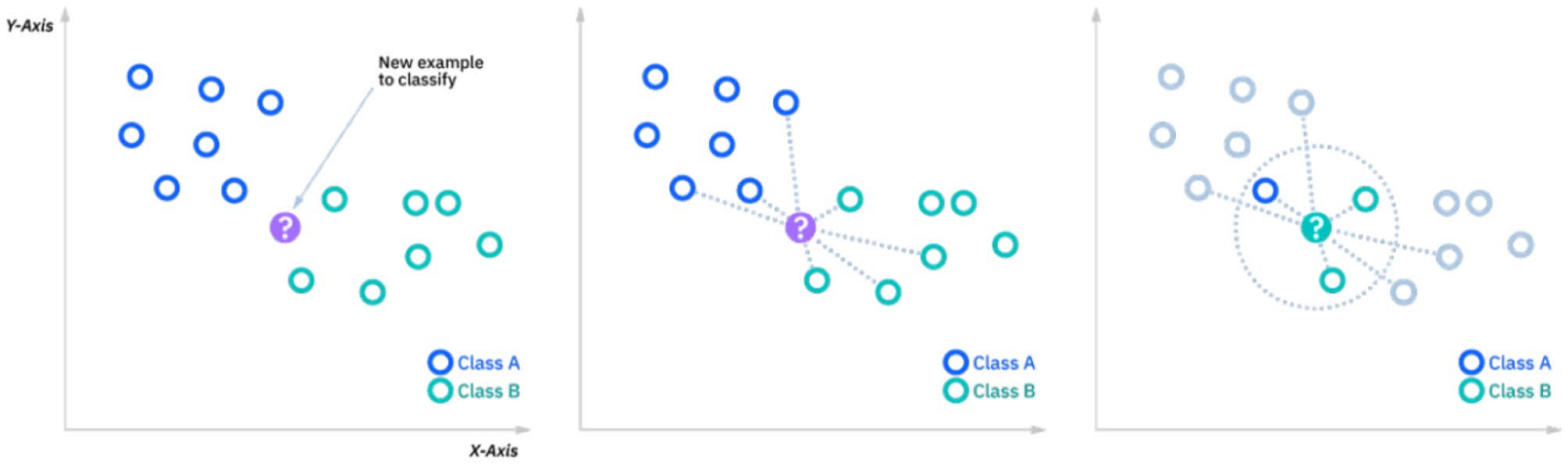
KNN.

We classified the deep CNN features via SVM and KNN classifiers in this phase. KNN has gained much popularity in the research community in classification and regression tasks since it outperforms many other existing classifiers due to its simplicity and robustness. KNN calculates the distance between a test sample (k) with its neighbours and then groups the k test sample to its nearest neighbour. The KNN classifier is shown in Fig. 6

**Figure 7:**
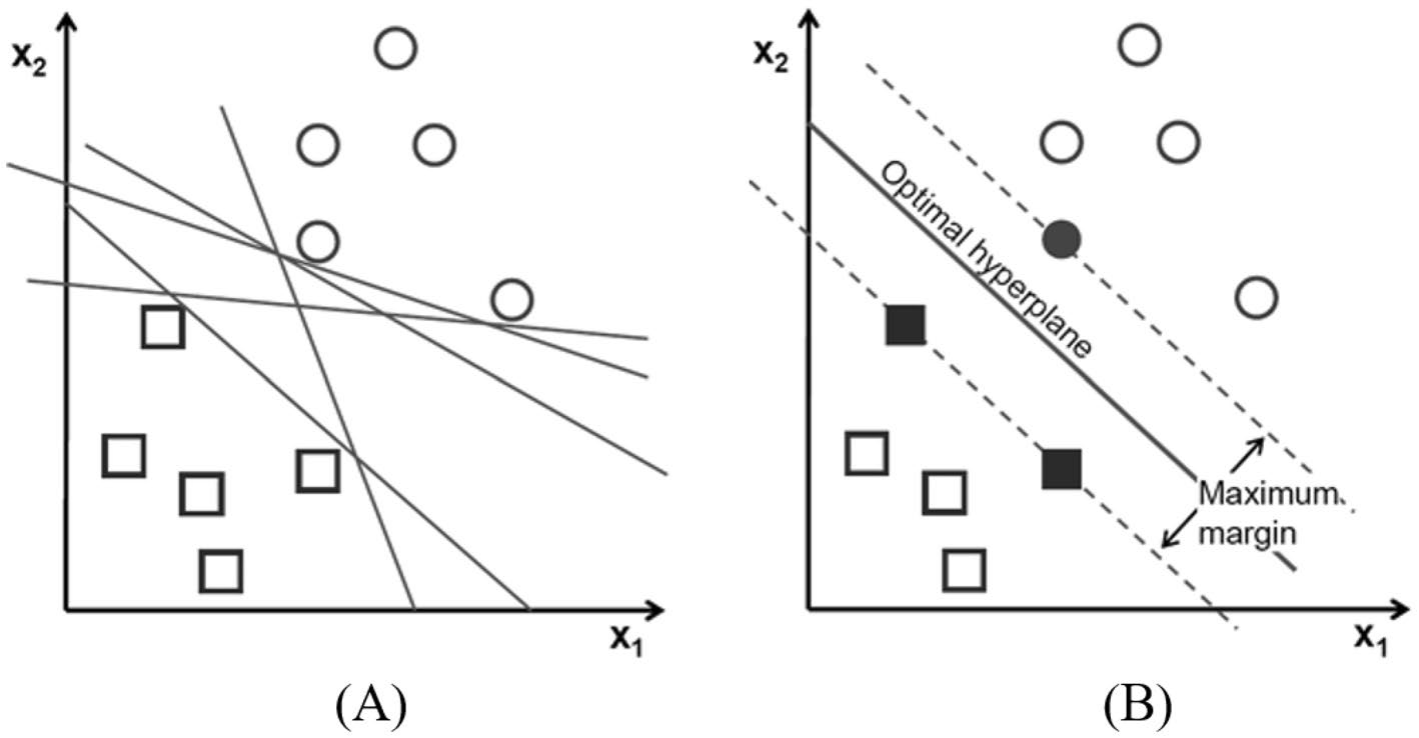
Possible SVM hyperplanes^30^.

The second classifier used in this study is SVM, a widely popular classifier used frequently in many research fields because of its faster speeds and superior prediction outcomes even on a minimal dataset. The classifier finds the plane with the largest margin that separates the two classes. The wider the margin better is the classification performance of the classifier^30,47^. Figure 7A depicts potential hyperplanes for a particular classification problem, whereas Fig. 7B depicts the best hyperplane determined by SVM for that problem.

## Results and discussion

### Dataset

**Figure 8:**
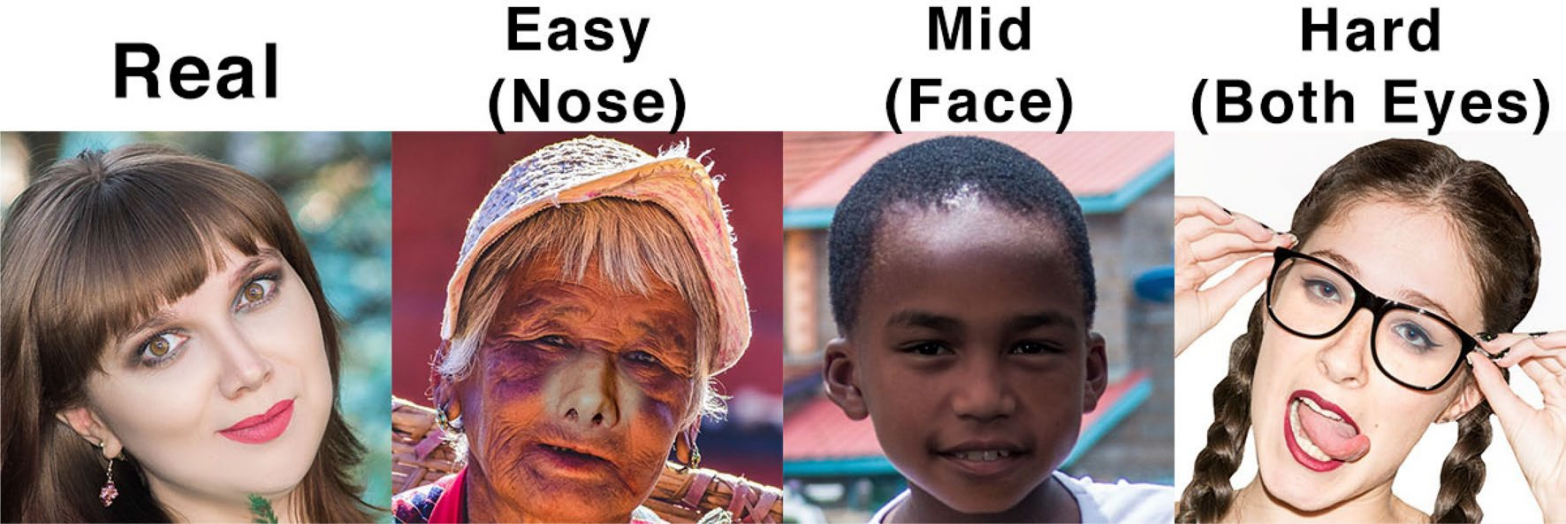
Image samples from the dataset showing real and edited images.

This study uses a publicly accessible dataset compiled by Yonsei University's Computational Intelligence and Photography Lab. The real and fake face database from Yonsei University's Computational Intelligence and Photography Lab is a dataset that contains images of both real and fake human faces. The dataset was designed for use in the research and development of facial recognition and verification systems, particularly those designed to detect fake or manipulated images. Each image in the dataset is labelled as either real or fake, and the dataset also includes additional information about the image, such as the age, gender, and ethnicity of the subject, as well as the manipulation technique used for fake images. Moreover, the images contain different faces, split by the eyes, nose, mouth, or entire face. The manipulated images further subdivided into three categories: easy, mid, and hard images as shown in Fig. 8^48^.

### Evaluation metrics

Evaluation metrics are used in machine learning to measure the performance of a model. Machine learning models are designed to learn from data and make predictions or decisions based on that data. It is important to evaluate the performance of a model to understand how well it is performing and to make necessary improvements. One of the most commonly used techniques is a confusion matrix, a table to evaluate the performance of a classification model by comparing the actual and predicted classes for a set of test data. It is a matrix of four values: true positives (TP), false positives (FP), true negatives (TN), and false negatives (FN). The proposed framework is evaluated using accuracy, precision, recall, and f1-score. Even though accuracy is a widely used metric, but is suitable in the case of a balanced dataset; hence, we also evaluated our proposed methods using F1-Score that combines both recall and precision into a single metric. All the evaluation metrics that we used to assess our models are calculated from Eq. (1) to Eq. (4).

1 $${\mathrm ACC }=\frac{{\mathrm TN}+{\mathrm TP}}{{\mathrm TP}+{\mathrm FN}+{\mathrm TN}+{\mathrm FP}}$$

2 $${\mathrm REC}=\frac{{\mathrm TP}}{{\mathrm FN}+{\mathrm TP}}$$

3 $${\mathrm PRE }=\frac{{\mathrm TP}}{{\mathrm FP}+{\mathrm TP}}$$

4 $${\mathrm F}1-{\mathrm Score }=\frac{{\mathrm TP}}{{\mathrm TP}+\frac{1}{2 }(FP+FN)}$$

### Proposed method results

The escalating problems with deep fakes have made researchers more interested in media forensics in recent years. Deep fake technology has various applications in the media sector, including lip sync, face swapping, and de-aging humans. Although advances in DL and deep fake technology have various beneficial applications in business, entertainment, and the film industry, they can serve harmful goals and contribute to people's inability to believe what's true^49,50^. Hence, finding the difference between real and fake has become vital in this age of social media. Finding deep fake content via the human eye has become more difficult due to progress in deep fake creation technologies. Hence, a robust system must be developed to classify these fake media without human intervention accurately.

**Figure 9:**
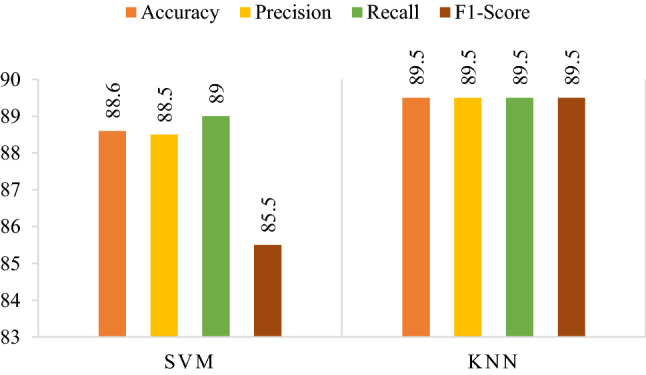
Results obtained from ResNet18's confusion matrix.

In this study, we propose a novel and robust architecture to detect and classify deep fake images using ML and DL-based techniques. The proposed framework employs a preprocessing approach to find ELA. ELA helps find if any portion of the image has been altered by analyzing the image on a pixel level. These images are then supplied to deep CNN architectures (SqueezeNet, ResNet18 & GoogLeNet) to extract deep features. The deep features are then classified via SVM and KNN. The results obtained from ResNet’s confusion matrix and ML classifiers is shown in Fig. 9. The feature vector achieved highest accuracy of 89.5% via KNN. We tested our various hyper-parameters for both classifiers before reaching the conclusion. The proposed method achieved 89.5% accuracy via KNN on Correlation as a distance metric and total 881 neighbors. SVM achieved 88.6% accuracy on Gaussian Kernel with a 2.3 scale.

**Figure 10:**
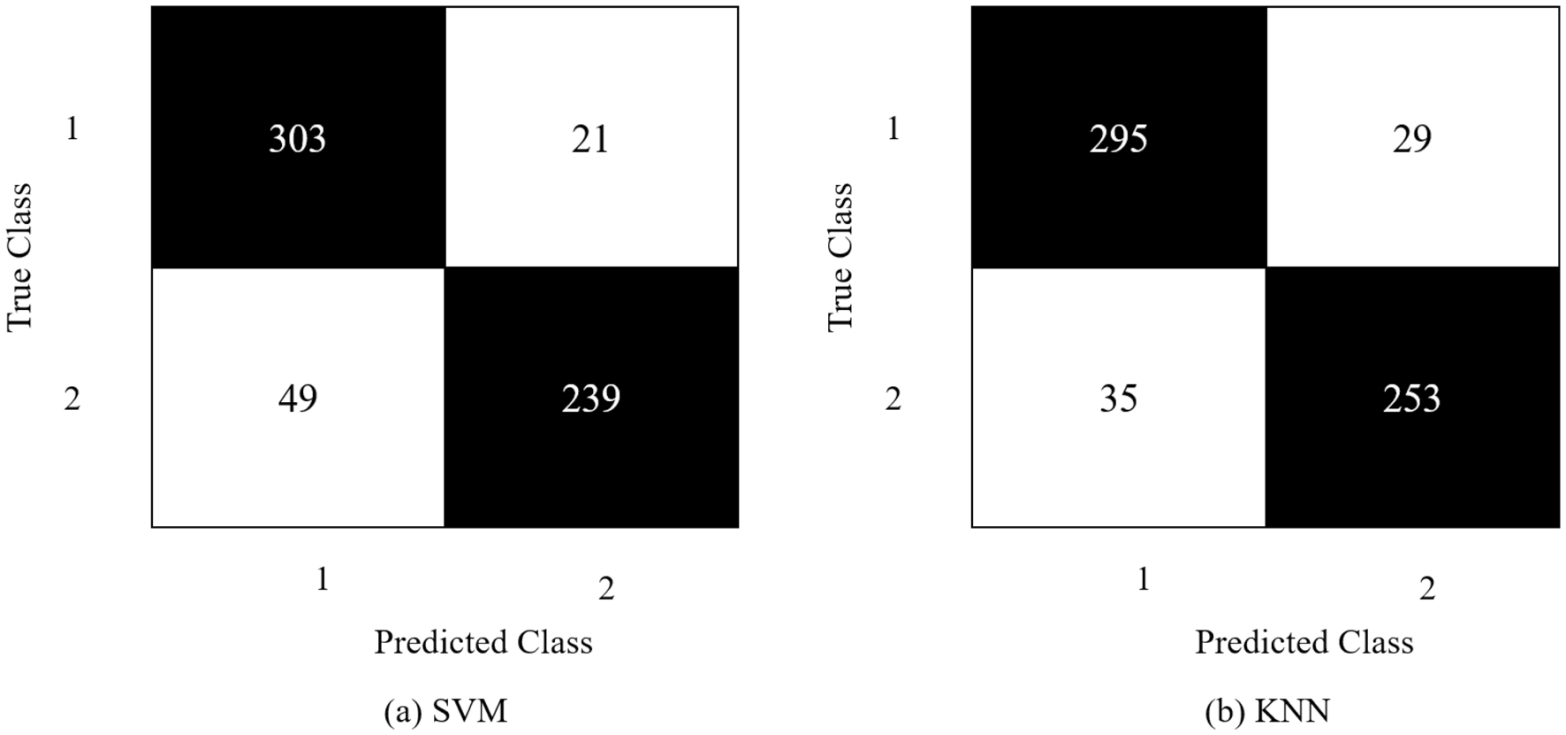
ResNet18's confusion matrix via (**a**) SVM, (**b**) KNN.

Hyperparameter optimization is the process of selecting the best set of hyperparameters for automated algorithms. Optimization is crucial for models because the model's performance depends on the choice of hyperparameters. We optimized parameters such as kernel functions, scale, no. of neighbors, distance metrics, etc., for KNN and SVM. The results obtained from the best parametric settings for different feature vectors are highlighted in bold text and shown in Table 1. Confusion matrices of both (a) SVM and (b) KNN are illustrated in Fig. 10.

**Table 1:** Hyper-parameter optimization on feature vector obtained from SVM and KNN.

SVM		KNN	
Hyper-parameters	Value/s	Hyper-parameters	Value/s
Kernel function	**Gaussian**, linear, quadratic, cubic	No. of neighbors	1–1021 (**881**)
Kernel scale	0.001–1000 (**2.4**)	Distance metric	City block, **correlation,** cosine, Euclidean, Hamming

**Figure 11:**
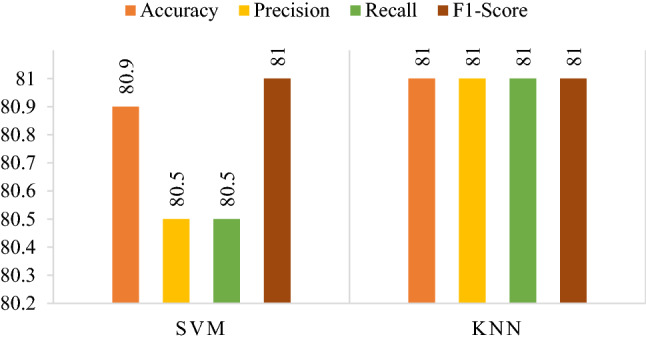
GoogLeNet’s results in terms of ACC, PRE, REC and F1-Score.

**Figure 12:**
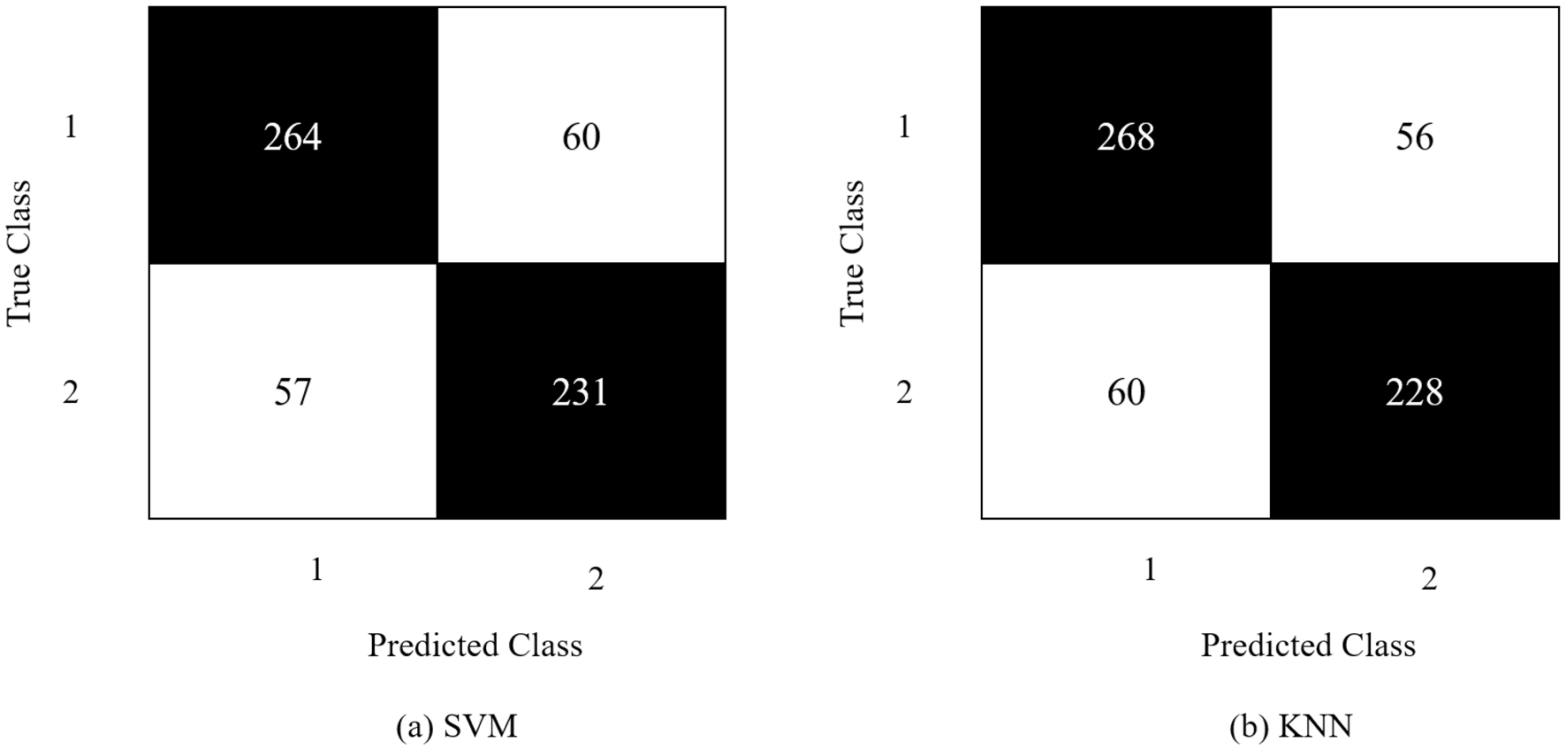
Confusion matrix obtained from GoogLeNet.

Moreover, the feature vector obtained from GoogLeNet’s obtained the highest accuracy of 81% via KNN on Chebyshev as a distance metric with a total number of 154 neighbours. The SVM classified the feature vector with 80.9% accuracy on Gaussian kernel with a 0.41 kernel scale. The tested and optimal metrics (highlighted in bold) are mentioned in Table 2. Detailed results in other evaluation metrics are mentioned in Fig. 11, whereas Fig. 12 shows its confusion matrices.

**Table 2:** Hyper-parameter optimization on GoogLeNet's feature vector.

SVM		KNN	
Hyper-parameters	Value/s	Hyper-parameters	Value/s
Kernel function	**Gaussian**, linear, quadratic, cubic	No. of neighbors	1–1000 **(154)**
Kernel scale	0.001–1000 **(0.4)**	Distance metric	City block, **correlation,** cosine, Euclidean, Hamming

**Figure 13:**
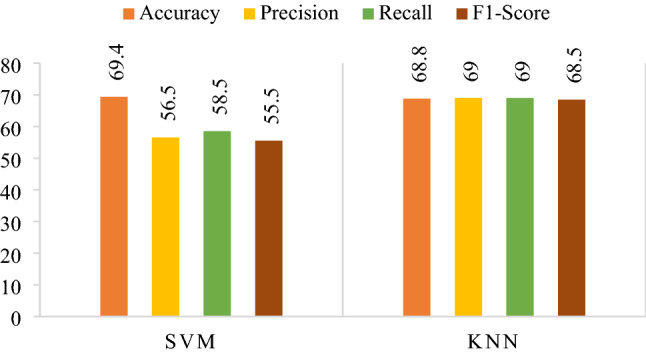
Results obtained from SqueezeNet's confusion matrices.

**Figure 14:**
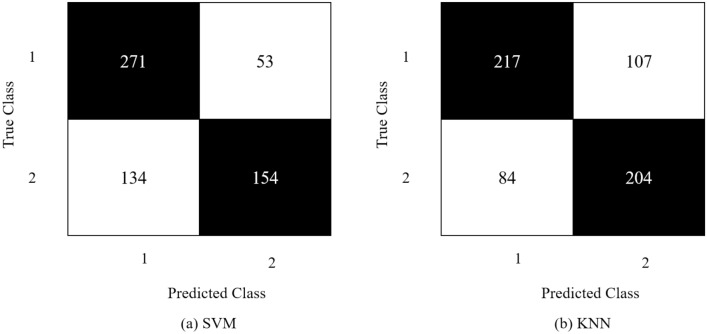
Confusion matrix obtained from SqueezeNet.

SVM and KNN classified the feature vector from SqueezeNet via 69.4% and 68.8%, respectively. The classifiers were evaluated on different parameters, as mentioned in Table 3 and achieved maximum performance on the parameters highlighted in bold text. The results in accuracy, precision, recall and f1-score are mentioned in Fig. 13. The confusion matrix is shown in Fig. 14.

**Table 3:** Hyper-parameter optimization on feature vector obtained from SqueezeNet.

SVM		KNN	
Hyper-parameters	Value (s)	Hyper-parameters	Value (s)
Kernel function	**Gaussian**, linear, quadratic, cubic	No. of neighbors	1–1000 **(154)**
Kernel scale	0.001–1000 **(0.4)**	Distance metric	City block, **correlation,** cosine, Euclidean, Hamming

### Comparison with state-of-the-art methods

This paper proposes a novel architecture to detect and classify deep fake images via DL and ML-based techniques. The proposed framework initially preprocesses the image to generate ELA, which helps determine if the image has been digitally manipulated. The resultant ELA image is then fed to CNN architectures such as GoogLeNet, ResNet18 and ShuffleNet for deep feature extraction. The classification is then performed via SVM and KNN. The proposed method achieved highest accuracy of 89.5% via ResNet18 and KNN. Residual Networks are very efficient and lightweight and perform much better than many other traditional classifiers due to their robust feature extraction and classification techniques. The detailed comparison is shown in Table 4. Mittal et al.^51^ employed Alex Net for deepfake detection. However, the study resulted in a very poor performance. Chandani et al.^50^ used a residual network framework to detect deep fake images. Similary, MLP and Meso Inception 4 by Matern et al.^15^ and Afchar et al.^26^ obtained more than 80% accuracy respectively. Despite being a deep CNN, Residual Networks perform much faster due to their shortcut connections which also aids in boosting the system’s performance. Hence, the proposed method performed much better on the features extracted from ResNet18.

**Table 4:** Comparison with state-of-the-art techniques.

Reference	Technique	Accuracy %
Mittal et al.^51^	AlexNet	55.8
Chandani et al.^52^	ResNet-152	76.7
Afchar et al.^26^	MesoInception4	81.3
Matern et al.^15^	MLP	85
Lee et al.^53^	Shallow Fake Face Net	72.5
Proposed	ResNet18 + KNN	89.5

## Conclusion

Deep faking is a new technique widely deployed to spread disinformation and hoaxes amongst the people. Even while not all deep fake contents are malevolent, they need to be found because some threaten the world. The main goal of this research was to discover a trustworthy method for identifying deep fake images. Many researchers have been working tirelessly to detect deep fake content using a variety of approaches. However, the importance of this study lies in its use of DL and ML based methods to obtain good results. This study presents a novel framework to detect and classify deep fake images more accurately than many existing systems. The proposed method employs ELA to preprocess images and detect manipulation on a pixel level. The ELA generated images are then supplied to CNNs for feature extraction. These deep features are finally classified using SVM and KNN. The proposed technique achieved highest accuracy of 89.5% via ResNet18’s feature vector & SVM classifier. The results prove the robustness of the proposed method; hence, the system can detect deep fake images in real time. However, the proposed method is developed using image based data. In the future, we will investigate several other CNN architectures on video-based deep fake datasets. We also aim to acquire real life deep fake dataset from the people in our community and use ML and DL techniques to distinguish between deep fake images and regular images to make it more useful and robust. It is worth mentioning that the ground-breaking work will have a significant influence on our society. Using this technology, fake victims can rapidly assess whether the images are real or fake. People will continue to be cautious since our work will enable them to recognize the deep fake image.

## Data Availability

The datasets used and/or analysed during the current study are available from the corresponding author on reasonable request.
